# Changing patterns of autochthonous malaria transmission in the United States: a review of recent outbreaks.

**DOI:** 10.3201/eid0201.960104

**Published:** 1996

**Authors:** J R Zucker

**Affiliations:** Centers for Disease Control and Prevention, Atlanta, Georgia 30333, USA. jxz2@ciddpd2.em.cdc.gov

## Abstract

Three recent outbreaks of locally acquired malaria in densely populated areas of the United States demonstrate the continued risk for mosquitoborne transmission of this disease. Increased global travel, immigration, and the presence of competent anopheline vectors throughout the continental United States contribute to the ongoing threat of malaria transmission. The likelihood of mosquitoborne transmission in the United States is dependent on the interactions between the human host, anopheline vector, malaria parasite, and environmental conditions. Recent changes in the epidemiology of locally acquired malaria and possible factors contributing to these changes are discussed.


### Changing Patterns of Autochthonous Malaria Transmission in the United States: A Review of Recent Outbreaks

**Jane R. Zucker, M.D., M.Sc.**
Centers for Disease Control and Prevention, Atlanta, Georgia, USA

Three recent outbreaks of locally acquired malaria in densely populated areas of the United States demonstrate the continued risk for mosquitoborne transmission of this disease. Increased global travel, immigration, and the presence of competent anopheline vectors throughout the continental United States contribute to the ongoing threat of malaria transmission. The likelihood of mosquitoborne transmission in the United States is dependent on the interactions between the human host, anopheline vector, malaria parasite, and environmental conditions. Recent changes in the epidemiology of locally acquired malaria and possible factors contributing to these changes are discussed.

Malaria was endemic throughout much of the United States in the late 19th and early 20th centuries (1). Interrupted human-vector contact, decreased anopheline populations, and effective treatment contributed to a decline in transmission and to subsequent eradication. However, environmental changes, the spread of drug resistance, and increased air travel (2) could lead to the reemergence of malaria as a serious public health problem. The potential for the reintroduction of malaria into the United States has been demonstrated by recent outbreaks of mosquitoborne transmission in densely populated areas of New Jersey, New York, and Texas (3-5). A review of the malaria life cycle and recent outbreaks illustrates key elements that affect the risk for malaria transmission in the United States.

### Life cycle and Entomologic Principles: Requirements for Transmission

The malaria parasites are protozoa of the genus *Plasmodium*. The four species of *Plasmodium* that cause human malaria, *P. falciparum, P. vivax, P. ovale, and P. malariae,* are transmitted by the bite of infective female mosquitoes of the genus *Anopheles*. The immature stages of the vector's life cycle (egg, larva, and pupa) are aquatic and develop in breeding sites, whereas the aerial adult stage is terrestrial. Anopheline species capable of transmitting malaria are found in all 48 states of the contiguous United States (1). The most important vectors are *An. quadrimaculatus* and *An. freeborni*, found east and west of the Rocky Mountains, respectively. However, other anopheline species have been implicated in local transmission, for example, *An. hermsi* in California (6).

Humans are the intermediate host and reservoir of the parasite, and the mosquito is the definitive host and vector. Female anophelines become infected only if they take a blood meal from a person whose blood contains mature male and female stages (gametocytes) of the parasite. A complex cycle of development and multiplication then begins with union of the male and female stages in the stomach of the vector and ends with parasites, called sporozoites, in its salivary glands, which are infective to humans (Figure 1). The time required for the complete maturation of the parasite (sporogonic cycle) in the mosquito varies and depends on the *Plasmodium* species and external temperature. At 27°C, approximately 8 to 13 days are needed for the completion of this cycle for *P. vivax* and *P. falciparum* (7). At lower temperatures, the time for the sporogonic cycle is considerably longer: approximately 20 days at 20°C and 30 days at 18°C for *P. vivax*. Similarly, for *P. falciparum*, the sporogonic cycle takes 30 days at 20°C. At a temperature below 16°C or 18°C, for these two species, respectively, the cycle cannot be completed and transmission cannot occur. On the other hand, 33°C is the upper limit for completion of the sporogonic cycle.

Only anophelines surviving longer than the sporogonic cycle can transmit malaria, assuming they took an infective blood meal. Extrinsic factors that affect the lifespan of the female anopheline,

Address for correspondence: Jane R. Zucker, Centers for Disease Control and Prevention, 1600 Clifton Road, MS F22, Atlanta, GA 30333; fax: 770-488-7761; e-mail:jxz2@ciddpd2.em.cdc.gov.

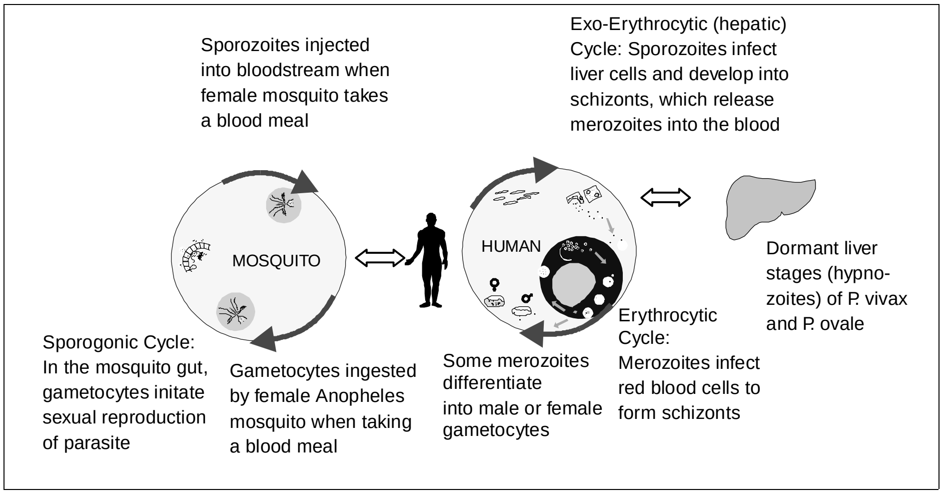

Figure 1. The malaria transmission life cycle.

and thus the completion of the sporogonic cycle, include ambient temperature, humidity, and rainfall. The efficiency and potential for transmission have been mathematically correlated to the survival of the mosquito population. Methods to determine the age range of mosquito populations are imprecise. Thus, determining the proportion of anophelines that have lived long enough to complete the sporogonic cycle is difficult.

Anophelines feed at night; therefore, transmission occurs primarily between dusk and dawn. When an infected mosquito takes a blood meal, it injects sporozoites from its salivary glands into the bloodstream (Figure 1). The sporozoites infect hepatocytes and begin a process of development and multiplication. The life cycle is completed when an anopheline takes a blood meal and ingests male and female gametocytes, allowing for sexual reproduction.

*P. vivax* gametocytes develop within the first few days of infection, and so a person may be infective early in the course of the illness. In contrast, *P. falciparum* gametocytes do not appear for a minimum of 10 to 14 days, by which time many people would have been symptomatic and received treatment. In addition, both *P. vivax* and *P. ovale* may form dormant liver stages, called hypnozoites, which may become active and cause a relapse of the infection and gametocytemia months to years after a person has left a malaria-endemic area. Hypnozoites are only formed at the time of the initial sporozoite inoculation.

This review of the malaria life cycle identifies the three factors essential for malaria transmission: adequate breeding sites and sufficient abundance of anophelines, weather conditions that allow completion of the sporogonic cycle, and gametocytemic persons. Historically, adequate housing, water management, and mosquito control activities acted to limit anopheline populations and prevented anopheline-human contact. In addition, conditions that promote mosquito survival and parasite development are not usually sustained; hence, the balance of these factors does not favor transmission. However, recent outbreaks demonstrate how tenuous the balance among these factors is. Changes that effect human-vector contact and increased density of gametocytemic persons during optimal weather conditions may be all that is necessary for transmission.

### Malaria Surveillance

### Historical Background

It is believed that malaria was introduced into the continental United States by European colonists (*P. vivax* and *P. malariae*) and African slaves (*P. falciparum*) in the 16th and 17th centuries. It became endemic in many areas of the country, paralleling the migration of people, with the exception of northern New England and mountainous and desert areas (Figure 2). The incidence of malaria probably peaked in approximately 1875, and it is estimated that more than 600,000 cases occurred in 1914 (1). Systematic reporting of malaria cases began in 1933; in 1934 125,556 cases were reported. The decline in transmission before the introduction of extensive mosquito control measures was attributed to a population shift from rural to urban areas, climatic conditions, increased drainage, improved housing and nutrition, better socioeconomic conditions and standards of living, greater access to medical services, and the availability of quinine for treatment (1). Additional activities, conducted in the 1940s, that led to the interruption of malaria transmission included larviciding, screening of houses, house spraying (residual spray program with DDT), and use of DDT (for residual spray and larviciding), which removed breeding sites, decreased the density of anophelines, and interrupted anopheline-human contact. Improved surveillance allowed treatment of parasitemic persons, focused control activities geographically, and allowed accurate assessment of the problem.

Surveillance was conducted by CDC to evaluate the progress toward malaria eradication, and in the 1950s it was concluded that this goal had been achieved. At that time, it was recognized that because of international travel, presence of competent anopheline vectors, and environmental conditions that could favor transmission, malaria could be reintroduced into the United States. Surveillance activities have been maintained not only to identify outbreaks of local malaria transmission, but also to identify other cases acquired in the United States (for example, transfusion-induced cases) and to monitor trends in imported cases that guide CDC prevention recommendations.

Since 1957, nearly all cases of malaria diagnosed in the United States have been imported, i.e., have been acquired by mosquito transmission (autochthonous) in areas where malaria is known to occur (8). In general, approximately half the cases occur among U.S. civilians and half among foreign-born civilians. However, each year cases occur that are acquired congenitally or are induced, i.e., acquired through artificial means, such as blood transfusions. Rarely, cases occur that are classified as cryptic (an isolated case of malaria determined after an epidemiologic investigation not to be associated with secondary cases) or introduced (a case documented to be acquired by mosquito transmission from an imported case in an area where malaria does not normally occur) (8). In practice, the distinction between a cryptic and an introduced case may be difficult to ascertain. Frequently, epidemiologic investigations indicate that the infection must have been acquired in the United States and circumstantial evidence suggests it was mosquitoborne. Additional evidence to document mosquitoborne transmission in the United States, such as the presence of anopheline larvae or infective adults, or confirmation of secondary transmission is rarely obtained.Therefore, all locally acquired cases thought to be mosquitoborne will be included in the following discussion, regardless of whether the final classification was cryptic or introduced.

### Overview of Locally Acquired Cases

From 1957, when the current surveillance system began, through 1994, 76 cases of introduced and cryptic malaria were reported (9-27). Single cases in Louisiana in 1983 and in Massachusetts in 1985 involved patients who had recently received blood transfusions (28, 29). The infections were likely induced by transfusion, although they were classified as cryptic because serologic testing of available donors did not implicate a source person. Apart from these two, 74 cases were reported from 21 states, including three northern states (Oregon, north-central New York, and New Hampshire), that were probably acquired by mosquitoborne transmission in the United States (Figure 3). The most common species identified was *P. vivax*, which accounted for 59 (80%) cases; *P. malariae* accounted for six (8%) cases, and *P. falciparum* for five (7%); the species was not identified for the remaining four (5%) cases. In 1992, *P. vivax*, *P. falciparum*, *P. malariae*, and *P. ovale* were identified in 51%, 33%, 4%, and 3% of

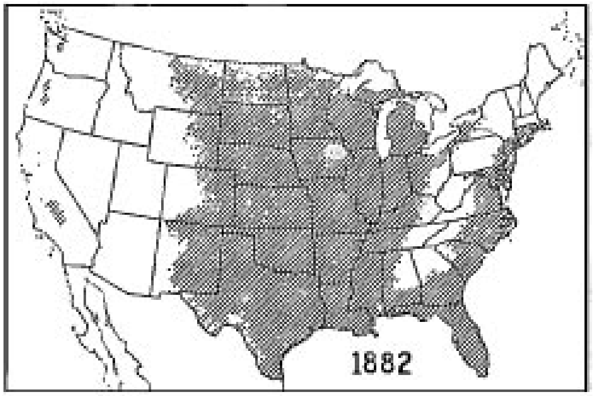

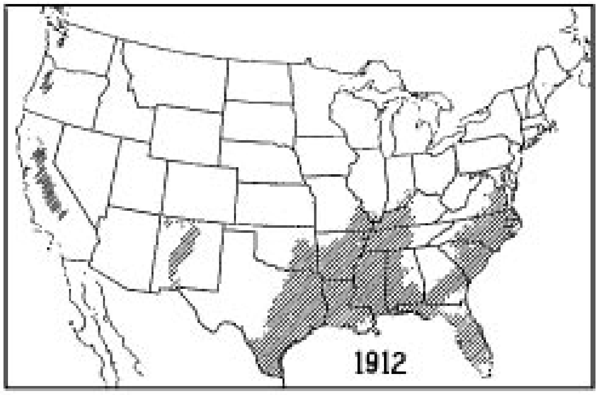

Figure 2. Areas of the United States where malaria was thought to be endemic in 1882 and 1912.

reported cases, respectively (30). The species was not identified in the remaining 9% of cases. The high proportion of locally acquired cases caused by *P. vivax* is not surprising for several reasons: vivax malaria is diagnosed most often among reported cases; the appearance of gametocytes early in the course of infection may allow for transmission to mosquitoes before treatment is received; relapse may occur months to years after leaving a malaria-endemic area when hypnozoites are reactivated; and the temperatures required for the completion of the sporogonic cycle are found in the United States.

The 74 cases represent 56 distinct episodes of probable transmission; 43 episodes involved one person without risk factors for malaria, nine involved two persons without risk factors, and four involved three or more persons. Before 1991, among cases with sufficient information, 41 (89%) of 46 outbreaks occurred in locations described as rural. Only three were in areas described as suburban, and two were in army barracks. Since then, the three episodes in New Jersey (1991), New York (1993), and Texas (1994) have all occurred in densely populated suburban or urban areas.

### California

From 1980 through 1990, 13 outbreaks of presumed mosquitoborne transmission were reported from California. Most occurred in rural areas where medical services were limited and sanitary facilities and housing were often substandard, allowing for anopheline-human contact; they involved undocumented migrant workers from malaria-endemic areas who were implicated as the gametocytemic source. During an outbreak in Carlsbad, California, in 1986 (6), 28 cases (26 Mexican migrant workers and two Carlsbad residents) of *P. vivax* were documented during a 3-month period. The epidemic curve indicated secondary transmission, thus confirming mosquitoborne transmission. The principal risk factor for malaria was sleeping on a particular hillside outdoors during the evening. Adult female anophelines (*An. hermsi*) were captured from a marsh area below the hillside, and temperature and humidity were favorable for completion of the sporogonic cycle.

### New Jersey

In 1991, two separate episodes of locally acquired *P. vivax* malaria were identified, occurring more than 70 miles apart (3); the first was consistent with the expected epidemiologic pattern, but the second occurred in a suburban and densely populated area. The index case-patient was an 8-year-old boy, without risk factors for malaria, (travel or exposure to blood or blood products). Few undocumented agriculture workers were living in this suburban area, but a large number of documented immigrants and undocumented factory workers were identified. U.S. census data from 1990 indicated that the population of immigrants from the Indian subcontinent,

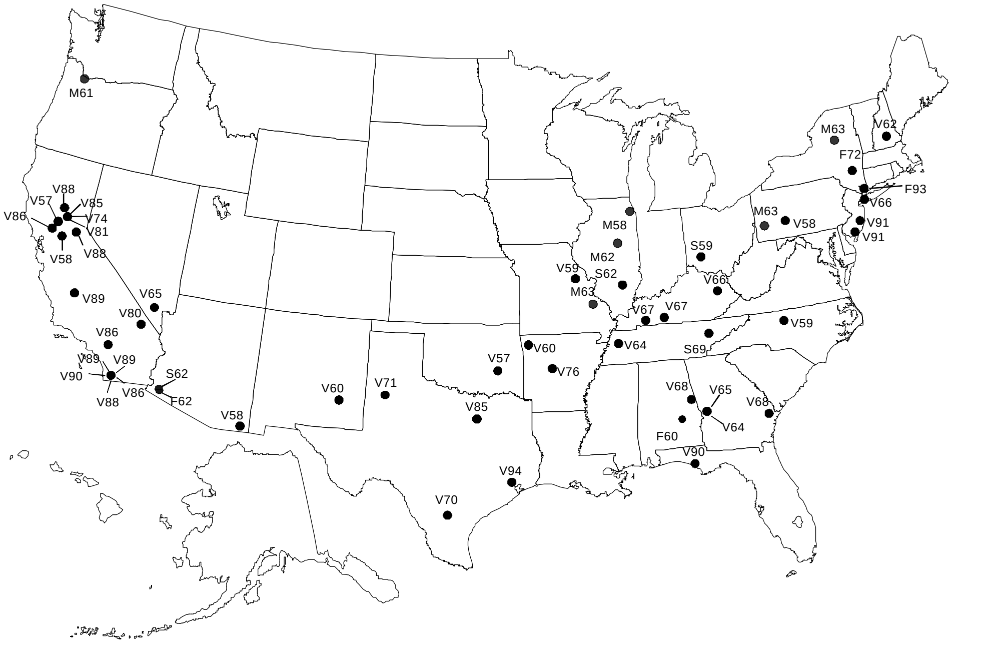

Figure 3. Location of presumed mosquitoborne malaria cases reported from 1957-1994. Each point denotes the location of the episode, the species identified (V = *Plasmodium vivax*, F = *P. falciparum*, M = *P. malariae* and *S = P. sp.*), and year of occurrence.

where malaria is endemic, increased by 230% compared with census data from 1980. The weather was hotter and more humid than usual, and higher anopheline densities were reported from some regions of New Jersey. The second case-patient had no clear exposure to mosquitoes but may have been exposed during the early evening in a marshy area where he played ball.

### *New York City*

In 1993, another outbreak of locally acquired malaria occurred in New York City (4). The index patient had no travel history or other means of acquiring malaria except local mosquitoborne transmission. The investigation identified two other cases of malaria; one in a person who had traveled internationally 2 years previously, and a third case which was initially unreported. This outbreak was unusual, not only because urban areas are poor habitats for anophelines, but also because the causative parasite was *P. falciparum*. The area where the cases were identified in northwest Queens had many immigrants; the 1990 census showed a 31% increase in the number of foreign-born persons, which accounted for 48% of all recent immigrants into Queens. Many of these immigrants were from malaria-endemic areas, including parts of South and Central America and Hispaniola (Dominican Republic and Haiti). In addition, more than 100 cases of imported malaria were reported in New York City during 1993 (Malaria Section/Division of Parasitic Diseases/CDC unpublished surveillance data). As seen with the earlier outbreaks, the weather that summer was hotter and more humid than usual. During the several weeks between the proposed dates of transmission and the investigation, the weather had changed, interfering with the identification of active anopheline breeding sites or adult anophelines.

### *Houston, Texas*

Three cases of locally acquired malaria were identified in Houston in 1994 (5). This investigation had features similar to those seen in previous outbreaks in California. All three patients were homeless and lived in substandard housing, which provided an opportunity for exposure to anophelines at night. Two of the patients became ill, and malaria was diagnosed in July; the duration of illness was 11 days to 3 weeks. The third patient had symptoms in late July, but a diagnosis of malaria was not made until December when he had a relapse of *P. vivax*, which could only occur from mosquitoborne transmission. Results from an indirect immunofluorescence assay for malaria antibodies conducted on serum specimens obtained in August and December provided additional evidence that his illness during the summer was malaria. The infected persons lived in areas with large immigrant populations. Environmental investigation identified possible breeding sites, and adult female *An. quadrimaculatus* were captured in light traps. In addition, the average temperature and humidity favored mosquito survival and development.

The three outbreaks that occurred in the early 1990s in densely populated areas occurred in neighborhoods with many immigrants from countries with malaria transmission and weather that was hot and humid and, therefore, conducive to the completion of the sporogonic cycle and the survival of adult female anophelines. The delay between mosquito inoculation, diagnosis, and investigation often meant changes in weather and inability to confirm the presence of adult anophelines and active breeding sites.

### Discussion

Understanding the factors that contributed to these outbreaks and improving case surveillance will facilitate detection of future outbreaks and development of appropriate prevention and control measures.

Two necessary criteria must be met for malaria transmission: anopheline vectors capable of transmitting malaria and gametocytemic persons. Both exist throughout the United States. Under current conditions, the average lifespan of anophelines in the United States is less than the duration of the sporogonic cycle. A common feature of all recent outbreaks has been weather that is hotter and more humid than usual, which may increase anopheline survival and decrease the duration of the sporogonic cycle enough to allow for the development of infective sporozoites. The possible effect of weather on malaria transmission has been cited in recent articles on the potential consequences of global environmental changes (31-33).

Detection of locally acquired cases depends on accurate diagnosis and reporting of cases. Prompt reporting is not universal as suggested by the Houston investigation (5). Delays in recognizing cases are caused by not suspecting malaria in a person with a febrile illness who has not traveled internationally, by laboratories inexperienced with blood smear diagnosis, and by general lack of reporting of notifiable diseases. Prompt diagnosis, treatment, and notification are essential for proper treatment and evaluation of potentially gametocytemic persons.

Alternative hypotheses for explaining malaria infection acquired in areas without ongoing transmission have included importation of infective anophelines either on airplanes, ships, or in baggage (34-36). One recent report of two persons who acquired *P. falciparum* in Germany indicates that conditions supporting local mosquitoborne transmission were present in Germany, although the authors concluded that infected mosquitoes must have been imported in baggage (37). Like the United States, many parts of Europe, including regions of Germany, have had endemic malaria transmission and thus are at risk for introduced autochthonous transmission. These alternative hypotheses have been addressed in the U.S. investigations, but none of the episodes occurred close enough to international airports or harbors to support these hypotheses. The possibility of “baggage malaria” is intriguing but unlikely for reasons concerning mosquito survival during transport and expected host-seeking behavior once the mosquitoes are released from luggage.

Gametocytemic persons, both immigrants and native-born U.S. civilians, are present in the United States and can serve as reservoirs of infection. Water management, improved housing, and access to health care are critical for preventing transmission. Diligent malaria surveillance can detect outbreaks early and allow control measures to interrupt transmission.

### Acknowledgments

I thank Dr. Monica Parise for her efforts in conducting the literature review on locally acquired malaria in the United States, and Dr. Ray Beach for his thoughtful comments and review of the manuscript.

### References

1. Report for registration of malaria eradication from United States of America. Washington, DC: Pan American Health Organization, December 1969.
2. Lederberg J, Shope RE, Oaks SC, Jr., editors. Emerging infections: microbial threats to health in the United States. Washington, DC: Institute of Medicine, National Academy Press, 1992.
3. Brook JH, Genese CA, Bloland PB, Zucker JR, Spitalny KC. Malaria probably locally acquired in New Jersey. N Engl J Med 1994;331:22-3.
4. Layton M, Parise ME, Campbell CC, Advani R, Sexton JD, Bosler EM, Zucker JR. Malaria transmission in New York City, 1993. Lancet 1995;346:729-31.
5. Local transmission of *Plasmodium vivax* malaria—Houston, Texas, 1994. MMWR 1995;44:295-303.
6. Maldonado YA, Nahlen BL, Roberto RR, Ginsberg M, Orellana E, Mizrahi M, et al. Transmission of *Plasmodium vivax* malaria in San Diego County, California, 1986. Am J Trop Med Hyg 1990;42:3-9.
7. Pampana E. A textbook of malaria eradication. London: Oxford University Press, 1963.
8. World Health Organization. Terminology of malaria and of malaria eradication, 1963. Geneva, Switzerland: World Health Organization, 1963:32.
9. Dunn FL, Brody JA. Malaria surveillance in the United States, 1956-1957. Am J Trop Med Hyg 1959;3:447-55.
10. Shaw JD, Schrack WD, Jr. Malaria contracted in Pennsylvania. Public Health Rep 1966;81:413-8.
11. Luby JP, Schultz MG, Nowosiwsky T, Kaiser RL. Introduced malaria at Fort Benning, Georgia, 1964-1965. Am J Trop Med Hyg 1967;16:146-53.
12. Jacobs T. Cryptic malaria. Rocky Mountain Medical Journal 1966;63:57-9.
13. Steiner ML, Malaria in a Kentucky family: report of two cases in siblings. Clin Pediatr (Phila) 1968;7:493-4.
14. Sartoriano GP, Rowden RM, Ginsburg DM. Malaria acquired in the United States: introduced and cryptic malaria. NY J Med 1971;71:1535-7.
15. Hermos JA, Fisher GU, Schultz MG, Haughie GE. Case histories in 1968 outbreak in Chambers. J Med Assoc Ala. 1969;39:57-66.
16. Dover AS. A malaria outbreak in Texas, 1970. South Med J 1972;65:215-8.
17. Center for Disease Control. Introduced malaria in Texas. MMWR 1970;19:407-8.
18. Singal M, Shaw PK, Lindsay RC, Roberto RR. An outbreak of introduced malaria in California possibly involving secondary transmission. Am J Trop Med Hyg 1977;26:1-9.
19. Malaria in California [letter]. West J Med 1981; 134:645-6.
20. Centers for Disease Control. Introduced autochthonous vivax malaria—California, 1980-1981. MMWR 1982;31:213-5.
21. Centers for Disease Control. Transmission of *Plasmodium vivax* malaria—San Diego County, California, 1986. MMWR 1986;35:679-81.
22. Brillman J. *Plasmodium vivax* malaria from Mexico—a problem in the United States. West J Med 1987;147:469-73.
23. Ginsberg MM. Transmission of malaria in San Diego County, California. West J Med 1991;154:465-6.
24. State of California—Health and Welfare Agency. Mosquito-transmitted malaria in California:1988-1989: part 1. California Morbidity, December 22, 1989.
25. State of California—Health and Welfare Agency. Mosquito-transmitted malaria in California:1988-1989: part 2. California Morbidity, January 5, 1990.

26. Centers for Disease Control. Transmission of *Plasmodium vivax* malaria—San Diego County, California, 1988 and 1989. MMWR 1990;39:91-4.
27. Centers for Disease Control. Mosquito-transmitted malaria—California and Florida, 1990. MMWR 1991;40:106-8.
28. Centers for Disease Control. Malaria surveillance annual summary 1983. Atlanta, GA: Centers for Disease Control, October 1984.
29. Centers for Disease Control. Malaria surveillance annual summary 1985. Atlanta, GA: Centers for Disease Control, September 1986.
30. Zucker JR, Barber AM, Paxton LA, Schultz LJ, Lobel HO, Roberts JM, et al. Malaria surveillance—United States, 1992. MMWR 1995;44(SS-5):1-17.
31. Loevinsohn ME. Climatic warming and increased malaria incidence in Rwanda. Lancet 1994;343:714-7.
32. Haines A, Epstein PR, McMichael AJ. Global health watch: monitoring impacts of environmental change. Lancet 1993;342:1464-9.
33. Bouma MJ, Sondorp HE, van der Kaay HJ. Climate change and periodic epidemic malaria. Lancet 1994;343:1440.
34. Isaacson M. Airport malaria: a review. Bull World Health Organ 1989;67:737-43.
35. Delmont J, Brouqui P, Poullin P, Bourgeade A. Harbour-acquired *Plasmodium falciparum* malaria. Lancet 1994;344:330-1.
36. Castelli F, Caligaris S, Matteelli A, Chiodera A, Carosi G, Fausti G. "Baggage malaria" in Italy: cryptic malaria explained? Trans R Soc Trop Med Hyg 1993;87:394.
37. Mantel CF, Klose C, Scheurer S, Vogel R, Wesirow AL, Bienzle U. *Plasmodium falciparum* malaria acquired in Berlin, Germany. Lancet 1995;346:320-1.
